# The receptor genes *PfBMPR1B* and *PfBAMBI* are involved in regulating shell biomineralization in the pearl oyster *Pinctada fucata*

**DOI:** 10.1038/s41598-017-10011-y

**Published:** 2017-08-23

**Authors:** Shiguo Li, Yangjia Liu, Jingliang Huang, Aibin Zhan, Liping Xie, Rongqing Zhang

**Affiliations:** 1 Institute of Marine Biotechnology, School of Life Sciences, Tsinghua University, Beijing, 100084 China; 2 Research Center for Eco-Environmental Sciences, Chinese Academy of Sciences, Beijing, 100085 China; 3 Department of Biotechnology and Biomedicine, Yangtze Delta Region Institute of Tsinghua University, Jiaxing, 314006 China

## Abstract

Mounting evidence suggests that TGFβ/BMP signaling pathway is most likely involved in shell biomineralization in molluscs, but the function of pathway receptors is poorly studied. Here, we cloned and identified two homologous BMP receptor genes, *PfBMPR1B* and *PfBAMBI*, from the pearl oyster *Pinctada fucata*. Real-time quantitative PCR and *in situ* hybridization revealed that these genes were expressed in mantle edge and pallial, specifically located at the outer epithelia. Knockdown of *PfBMPR1B* by RNA interference (RNAi) significantly decreased the expression levels of matrix protein (MP) genes and induced the abnormal ultrastructure of prismatic and nacreous layers. Conversely, knockdown of *PfBAMBI* significantly increased the expression levels of a portion of MP genes and induced the overgrowth of nacreous layer crystals. In the RNAi and shell notching experiments, MP gene expressions were competitively regulated by *PfBMPR1B* and *PfBAMBI*. In addition, the receptor inhibitor LDN193189 reduced the expression levels of MP genes in mantle primary cells and larvae, and induced abnormal D-shaped shell formation during larval development. Collectively, these results clearly show that *PfBMPR1B* and *PfBAMBI* are involved in regulating shell biomineralization in *P*. *fucata*. Our study therefore provides the direct evidence that BMP receptors participate in mollusc biomineralization.

## Introduction

Biomineralization is a widespread process in which inorganic ions orderly accumulate and deposit on the intracellular or extracellular matrix under the guidance of biomacromolecules in living organisms^1^. Mollusc shells and pearls are the major biomineralization products in nature. In the past decades, shell biomineralization of molluscs has been extensively investigated owing to the attractive scientific, medicinal and commercial values of shells and pearls. These investigations revealed that matrix proteins (MPs), a kind of biomacromolecules secreted from the mantle, were primarily involved in controlling the deposition of shell mineral phase—calcium carbonate (CaCO_3_)^2–5^. An increasing number of genes encoding the MPs have been identified^6^ and the expression of these genes has been proposed to inevitably undergo a series of rigorous molecular regulations, promoting our understanding of the mechanism of mollusc biomineralization.

Signal regulation is an important molecular mechanism of biomineralization. As a functionally conserved pathway, the transforming growth factor beta (TGFβ) signaling pathway plays key roles in growth, development and reproduction throughout the life history of vertebrates and invertebrates^7, 8^. The bone morphogenetic protein (BMP) signaling pathway, a subgroup of TGFβ, is involved in bone and tooth biomineralization in vertebrates^9, 10^. This pathway has also received much attention with respect to its functions during shell formation. For example, studies on the adult stage of molluscs have shown that the members of the TGFβ/BMP signaling pathway, including Smads (Sma- and mad-related protein homologs)^11^, Bmps (bone morphogenetic proteins)^12, 13^ and Bmp receptors^14^, were expressed in the mantle, the calcifying organ of molluscs, of the pearl oyster *Pinctada fucata* and the Pacific oyster *Crassostrea gigas*. Knockdown of the *SMAD4*, *BMP2* and *BMP7* genes by RNAi resulted in disordered crystallization on the surface of the shell^11, 12^. At the larval stage, *SMAD4* and *SMAD1/5/8* were largely detected at the position of shell germination in the embryos of *C*. *gigas* ^15^. Meanwhile, analysis of the larvae of the brachiopod *Lingula anatina* revealed that *SMAD1/5/9* was activated at the anterior margin of the mantle lobe during larval shell formation^16^. All these findings propose a hypothesis that the TGFβ/BMP signaling pathway is highly likely to be associated with shell biomineralization. Although efforts have been made, the regulatory mechanism of mollusc biomineralization is far from being fully understood at the molecular level. Recent studies reported that the transcription factor genes *POU3F4* ^17^ and *MSX* ^18^ play important roles in biomineralization in pearl oysters by influencing transcriptional activities of the MP genes. Nevertheless, these transcription factors are nuclear effectors, and they generally work together with signaling transmitters (e.g., Smads) in specific signaling pathways. Study of the TGFβ/BMP signaling pathway may be one of the important ways to reveal the regulatory mechanism of shell biomineralization.

Receptors are the main members of the TGFβ/BMP signaling pathway^19, 20^. Bmpr1b is a type I receptor that interacts with a ligand-Bmpr2 complex, forming the heterologous tetramer Bmpr2-Bmpr1b. The glycine/serine-rich (GS) domain of Bmpr1b in the tetramer-complex in turn phosphorylates the downstream Smads, which move into the nucleus to regulate transcription of target genes^21^. Simultaneously, BMP and activin membrane-bound inhibitor (Bambi), a pseudoreceptor, can competitively bind with the ligands and Bmpr2, replacing the Bmpr1 position in the heterologous tetramer complex. Bambi is relatively short and lacks the intracellular GS domain required for phosphorylation, resulting in termination of the signal transduction^22^. Evidently, there is competitive regulation between Bmpr1 and Bambi in the TGFβ/BMP signaling pathway. It is well accepted that *BMPR1B* and *BAMBI* regulate bone biomineralization in vertebrates^23, 24^. In contrast to abundant findings in vertebrates, we have limited knowledge on the roles of receptors in mollusc biomineralization. Although related studies have successfully cloned and characterized *BMPR1*, *TGFR1*, *ACVRI*, *ACVRII*, and *ALR1* in the Pacific oyster *C*. *gigas* ^14, 25–27^, *ALR1* in the pearl oyster *P*. *fucata* ^28^ and *TGFR1* in the scallop *Chlamys farreri* ^29^, there was no direct evidence to support their roles in the biomineralization process. Therefore, it is largely needed to perform detailed investigation of BMP receptors in shelled molluscs to supply reliable data for the involvement of TGFβ/BMP signaling pathway in shell biomineralization.

The pearl oyster *P*. *fucata* is one of the model molluscs for the study of biomineralization. The draft genome^30^ and specifically sequenced transcriptome^31^ of this species provide abundant gene information regarding the members of the TGFβ/BMP signaling pathway. In the present study, we identified two receptor genes, *PfBAMBI* and *PfBMPR1B*, of the TGF-β/BMP signaling pathway from *P*. *fucata* and clarified their roles in shell biomineralization regulation. Multiple analyses, including *in situ* hybridization, reverse transcription quantitative real-time PCR, shell notching, RNA interference, and a yeast two-hybrid system, were employed to study the expression and location of *PfBAMBI* and *PfBMPR1B*, the interaction between these two receptors and other pathway members, and the effects of *PfBAMBI* and *PfBMPR1B* on MP gene expression and shell ultrastructure. The mantle primary cells and larval stage of *P*. *fucata* were exposed to the receptor inhibitor LDN193189 to further confirm the involvement of type I receptors, particularly *PfBMPR1B*, in shell biomineralization. These results will provide direct evidence regarding whether the BMP receptors participate in the regulation of shell biomineralization in pearl oysters and verify the hypothesis that TGFβ/BMP signaling pathway regulates mollusc biomineralization.

## Results

### Characterization and phylogenetic analysis of PfBMPR1B and PfBAMBI

**Figure 1:**
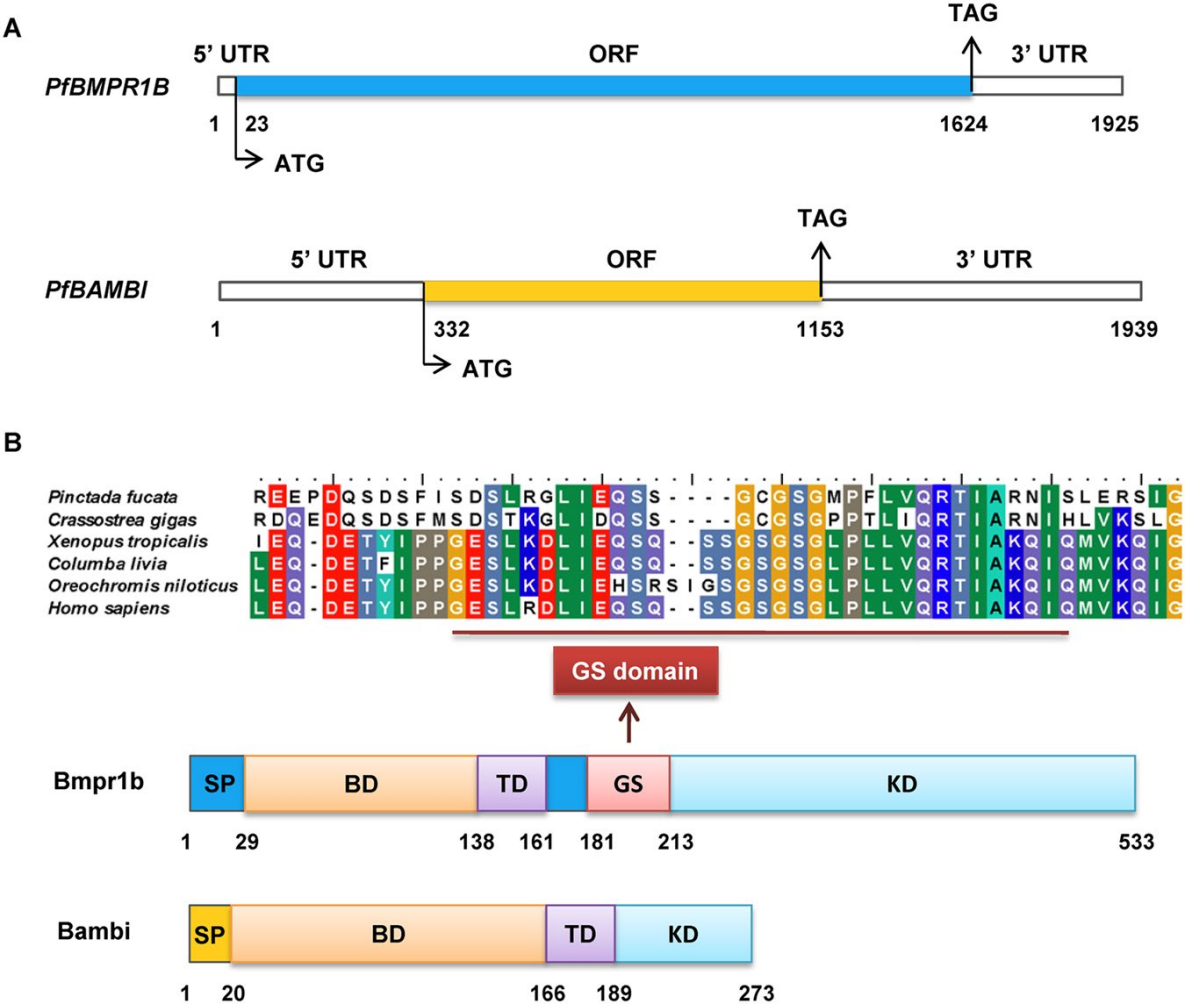
Schematic representations of gene structures (**A**) and deduced amino acid sequences (**B**) of *PfBMPR1B* and *PfBAMBI* in *Pinctada fucata*. (**A**) The blue and yellow boxes indicate the open reading frames (ORF) of *PfBMPR1B* and *PfBAMBI*, respectively. The upstream and downstream untranslated regions (5′UTR and 3′UTR) are represented as white boxes. ATG and TAG are initiation and termination codons, respectively. The lengths of the mRNA sequences are shown below the diagram. (**B**) The glycine/serine-rich domain (GS) is represented by sequence alignment with 6 species, including *Pinctada fucata* (GenBank accession number AGW51569.1), *Crassostrea gigas* (CAE11917.1), *Xenopus tropicalis* (NP_001072633.1), *Columba livia* (XP_005498077.1), *Oreochromis niloticus* (XP_003453955.2) and *Homo sapiens* (AAH47773.1). SP: Signal peptide. BD: Binding domain. TD: Transmembrane domain. KD: Kinase domain. The lengths of the amino acid sequences are shown below the diagram.

The full-length *PfBMPR1B* cDNA (GenBank accession number KF280238.1) is 1925 bp with a 5′ untranslated region (UTR) of 22 bp, a 3′UTR of 301 bp and an open reading frame (ORF) of 1602 bp (Fig. 1A). It encodes a protein of 533 amino acids (AA) with a predicted molecular mass of 60.75 kDa and an estimated isoelectric point of 5.94. Functional analysis revealed that the Bmpr1b protein has a signal peptide (SP) from 1 AA to 28 AA, a binding domain (BD) from 29 AA to 137 AA and a transmembrane domain (TD) located at the position ranging from 138 AA to 160 AA. A GS domain is predicted in the range of 181 AA to 212 AA (Figs 1B and S1). Twenty-three serine and threonine phosphorylation sites (kinase domain, KD) are also predicted and distributed at positions ranging from 213 AA to 521 AA.

The full-length *PfBAMBI* cDNA (GenBank accession number KF280237.1) is 1939 bp with a 5′UTR of 331 bp, a 3′UTR of 786 bp and an ORF of 822 bp (Fig. 1A). It encodes a protein of 273 AA with a predicted molecular mass of 30.51 kDa and an estimated isoelectric point of 8.82. The amino acid sequence from 19 AA to 109 AA deduced from the *PfBAMBI* ORF contains the specific features of a Bambi N-terminal domain (Figs 1B and S2). The putative SP cleavage occurs between 19 AA and 20 AA, releasing a 254-residue mature peptide. A BD is located from 21 AA to 165 AA. The hydrophobicity analysis suggests a potential TD between 166 AA and 188 AA. Six serine and threonine phosphorylation sites are predicted and distributed at positions ranging from 189 AA to 273 AA. Although it has a few phosphorylation sites, the amino acid sequence of *PfBAMBI* in *P*. *fucata* lacks an intracellular GS domain.

**Figure 2:**
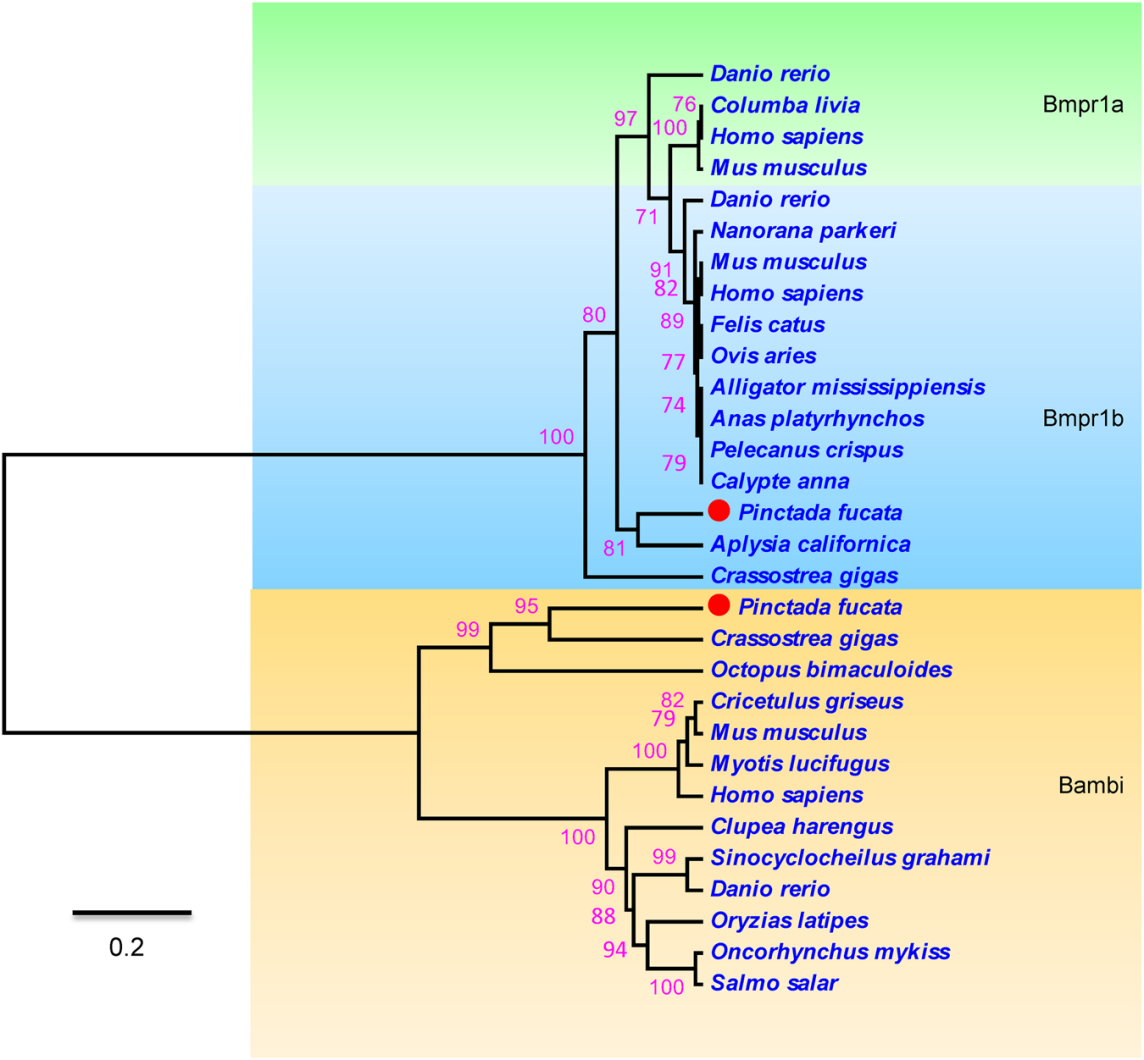
Phylogenetic tree based on amino acid sequences of *Bmpr1a*, *Bmpr1b* and *Bambi*. The GenBank accession numbers of different species in the tree are listed as follows: *Pinctada fucata* (AGW51569.1), *Crassostrea gigas* (CAE11917.1), *Octopus bimaculoides* (XP_014772317.1), *Clupea harengus* (XP_012670623.1), *Cricetulus griseus* (XP_016833151.1), *Myotis lucifugus* (XP_006086706.1), *Mus musculus* (NP_080781.1), *Oncorhynchus mykiss* (NP_001153956.1), *Salmo salar* (XP_014012981.1), *Sinocyclocheilus grahami* (XP_016085969.1), *Danio rerio* (AAH71292.1), *Homo sapiens* (BAD96785.1), *Oryzias latipes* (XP_004080931.1) and *Xenopus tropicalis* (NP_001008194.1) for Bambi; *Pinctada fucata* (AGW51569.1), *Crassostrea gigas* (CAE11917.1), *Lingula anatina* (XP_013392464.1), *Aplysia californica* (XP_005111811.1), *Calypte anna* (XP_008497826.1), *Pelecanus crispus* (XP_009491850.1), *Anas platyrhynchos* (XP_005024463.1), *Nanorana parkeri* (XP_018409998.1), *Ovis aries* (NP_001009431.1), *Alligator mississippiensis* (XP_014455760.1) and *Felis catus* (XP_006931018.1) for Bmpr1b; and *Danio rerio* (NP_571696.2), *Homo sapiens* (NP_004320.2), *Columba livia* (XP_005511641.1) and *Mus musculus* (AAH42611.1) for Bmpr1a. The red labels before the species name indicate the pearl oyster *P*. *fucata*.

A phylogenetic tree was constructed using the deduced amino acid sequences of *Bmpr1a*, *Bmpr1b* and *Bambi* in invertebrates and vertebrates (Fig. 2). The amino acids in different species are clustered into two separate branches (Bmpr1 branch and Bambi branch) in the phylogenetic tree. The Bmpr1 branch is further divided into two sub-branches, a Bmpr1a and a Bmpr1b sub-branches. The amino acids of *PfBAMBI* and *PfBMPR1B* are classified into the Bambi and Bmpr1b subbranch, respectively. They have high sequence similarity with the genes of the marine shelled mollusc *C*. *gigas* (50% and 63% identity, respectively). These results indicate that *PfBAMBI* and *PfBMPR1B* have a close relationship with the *BAMBI* and *BMPR1B* gene families, respectively.

### Bambi and Bmpr1b interact with members of the TGFβ/BMP pathway

**Figure 3:**
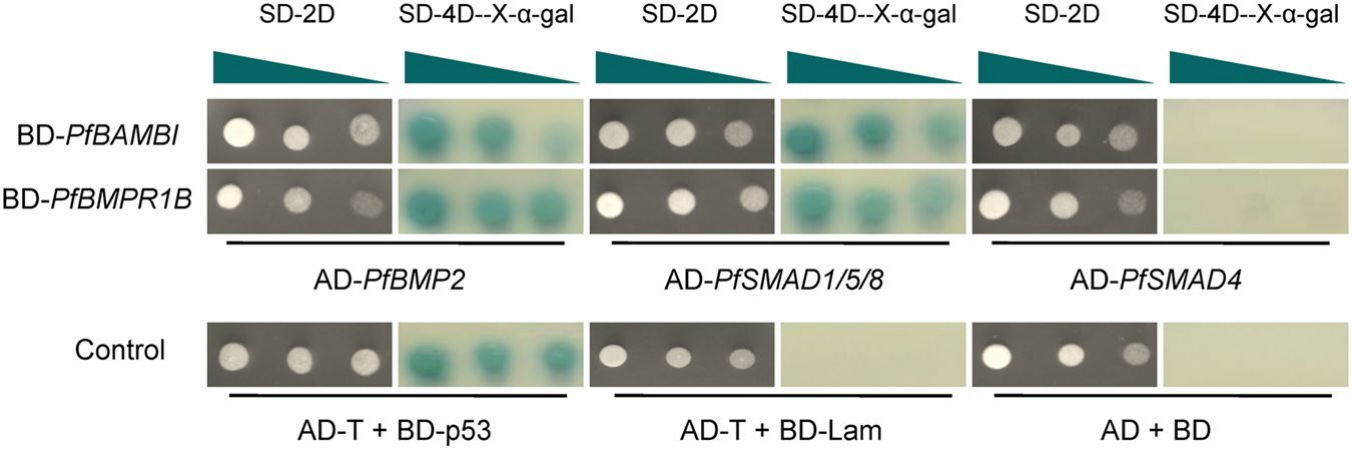
Interactions among Bambi, Bmpr1b and members of the TGFβ/BMP signaling pathway in *Pinctada fucata* detected by a yeast two-hybrid system. The plasmids are cotransformed into the yeast AH109 and spread on the dropout synthetically defined (SD) medium. The white colonies on SD-Leu-Trp medium (SD-2D) indicate that the plasmids are successfully transformed. The blue colonies on SD-Ade-His-Leu-Trp/X-α-Gal medium (SD-4D- X-α-Gal) indicate the presence of an interaction between two proteins, comparable with that of the positive (AD-T + BD-p53), negative (AD-T + BD-Lam) and empty plasmid (AD + BD) controls. The triangle indicates that the yeast is gradient diluted five times with sterilized water before spreading on the medium.

After incubating for 15 h, the OD_600_ values of the transformed yeast were greater than 0.8 (Supplementary Table 1). In addition, the transformed yeast grew well on the corresponding dropout mediums (Fig. S3). All these results indicated that the yeast two-hybrid (Y2H) system was efficient. As shown in Fig. 3, the white colonies grown on the synthetic dropout (SD) medium SD-Trp-Leu represent successful transformations of the plasmids pGBKT7 (BD)-*PfBAMBI*, BD-*PfBMPR1B*, pGADT7 (AD)-*PfBMP2*, AD-*PfSMAD1/5/8*, AD-*PfSMAD4* and AD-*PfBMPR1B*. The blue clones are observed for the yeast AH109 cotransformed with AD-*PfBMP2* and BD-*PfBAMBI*, AD-*PfSMAD1/5/8* and BD-*PfBAMBI*, AD-*PfBMP2* and BD-*PfBMPR1B* and AD-*PfSMAD1/5/8* and BD-*PfBMPR1B* on the SD-Trp-Leu-His-Ade/X-gal medium. However, no clones grew for the yeast cotransformed with AD-*PfSMAD4* and BD-*PfBAMBI* or AD-*PfSMAD4* and BD-*PfBMPR1B* on the SD-Trp-Leu-His-Ade/X-gal medium. The results indicate that Bmpr1b and Bambi interact with members of the TGFβ/BMP pathway in *P*. *fucata*.

### PfBMPR1B and PfBAMBI expression and location in mantle tissue

**Figure 4:**
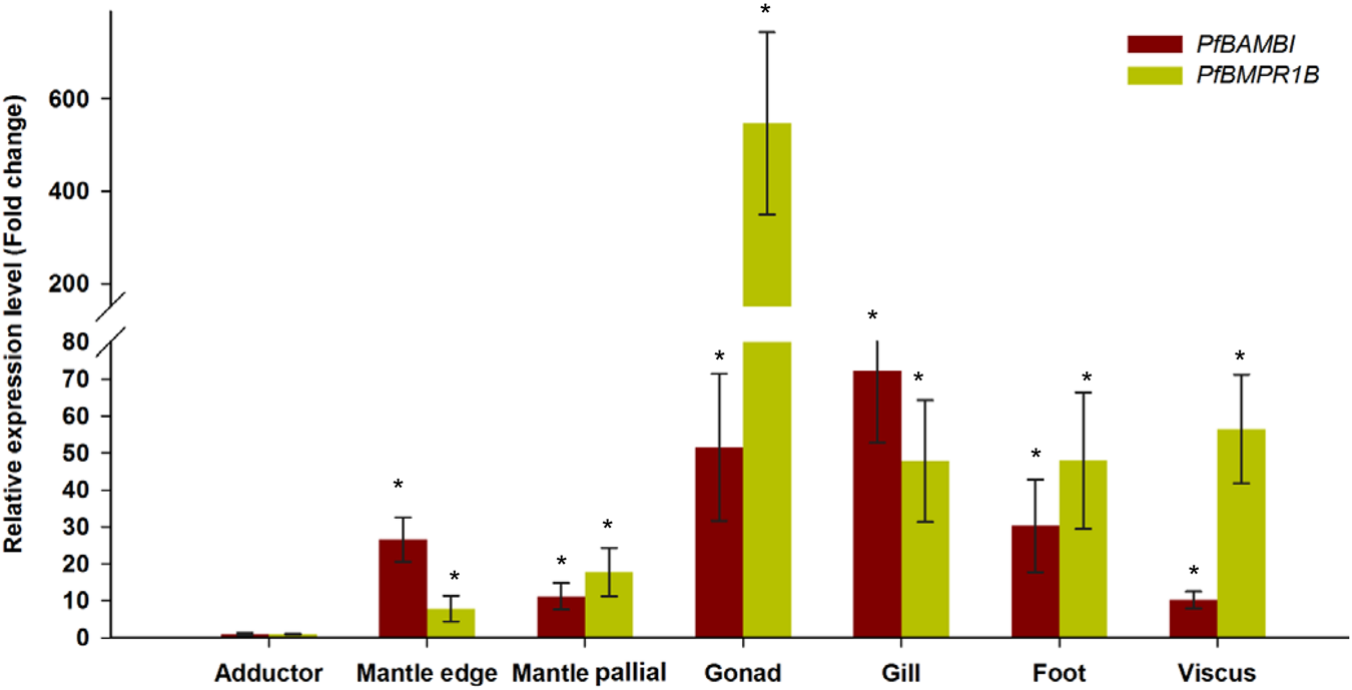
The relative expression levels of *PfBMPR1B* and *PfBAMBI* in different tissues of *Pinctada fucata*. Mantle edge, mantle pallial, gonad, gill, foot and viscus were selected as study tissues, and the expression levels of *PfBMPR1B* and *PfBAMBI* in the adductor were used as controls. *P < 0.05. Differences were assessed by one-way ANOVA.

**Figure 5:**
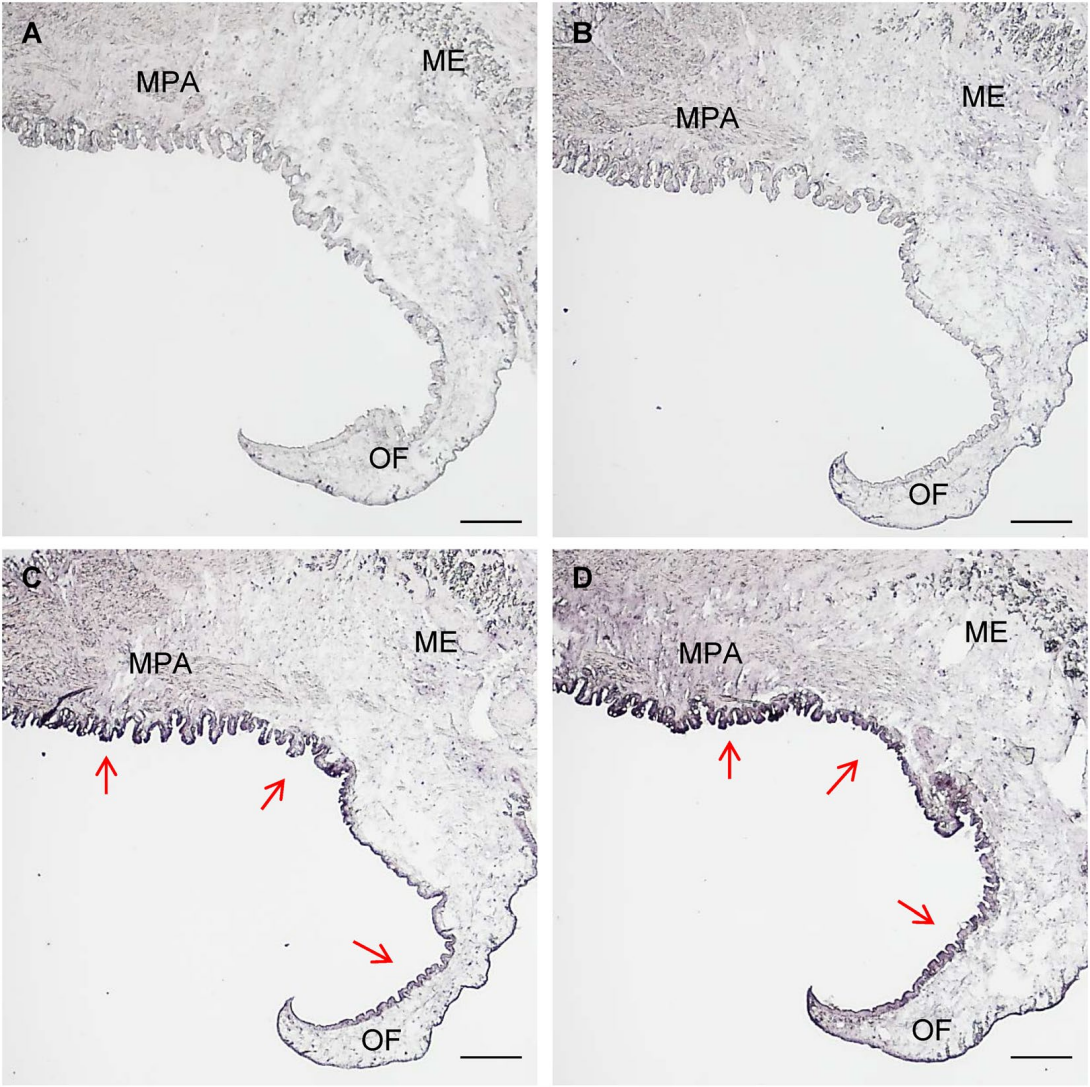
The localizations of *PfBMPR1B* and *PfBAMBI* in the mantle of *Pinctada fucata* detected by *in situ* hybridization. The sense probes for *PfBMPR1B* (**A**) and *PfBAMBI* (**B**) are used as controls. MPA: Mantle pallial. ME: Mantle edge. OF: Outer fold. The red arrows indicate the positive signals (dark blue) detected in the outer epithelia of the MPA and OF using antisense probes of *PfBMPR1B* (**C**) and *PfBAMBI* (**D**). Bar = 200 μm.

As shown in Fig. 4, the relative expression levels of *PfBAMBI* are 72.28-fold in gill, 51.59-fold in gonad, 30.28-fold in foot, 26.59-fold in mantle edge and 11.19-fold in mantle pallial higher than that in the adductor in *P*. *fucata*. For *PfBMPR1B*, they are 47.97-fold in gill, 547.00-fold in gonad, 47.96-fold in foot, 7.85-fold in mantle edge and 17.69-fold in mantle pallial higher than that in the adductor. The expression levels of *PfBMPR1B* and *PfBAMBI* in the mantle edge, mantle pallial, gonad, gill, foot and viscus are significantly higher than that in the adductor (*P* < 0.05), and the expression levels of *PfBMPR1B* and *PfBAMBI* in the gonad and gill are significantly higher than those in the mantle edge and mantle pallial (*P* < 0.05). Further investigation using *in situ* hybridization demonstrated that the detected signals for both *PfBMPR1B* and *PfBAMBI* are located in the outer epithelia of the mantle outer fold (OF) and the mantle pallial (Fig. 5C and D) compared with those in controls (Fig. 5A and B). All these findings imply that *PfBMPR1B* and *PfBAMBI* may be related to shell biomineralization in *P*. *fucata*.

### Pf-BMPR1B and PfBAMBI respond to shell notching

**Figure 6:**
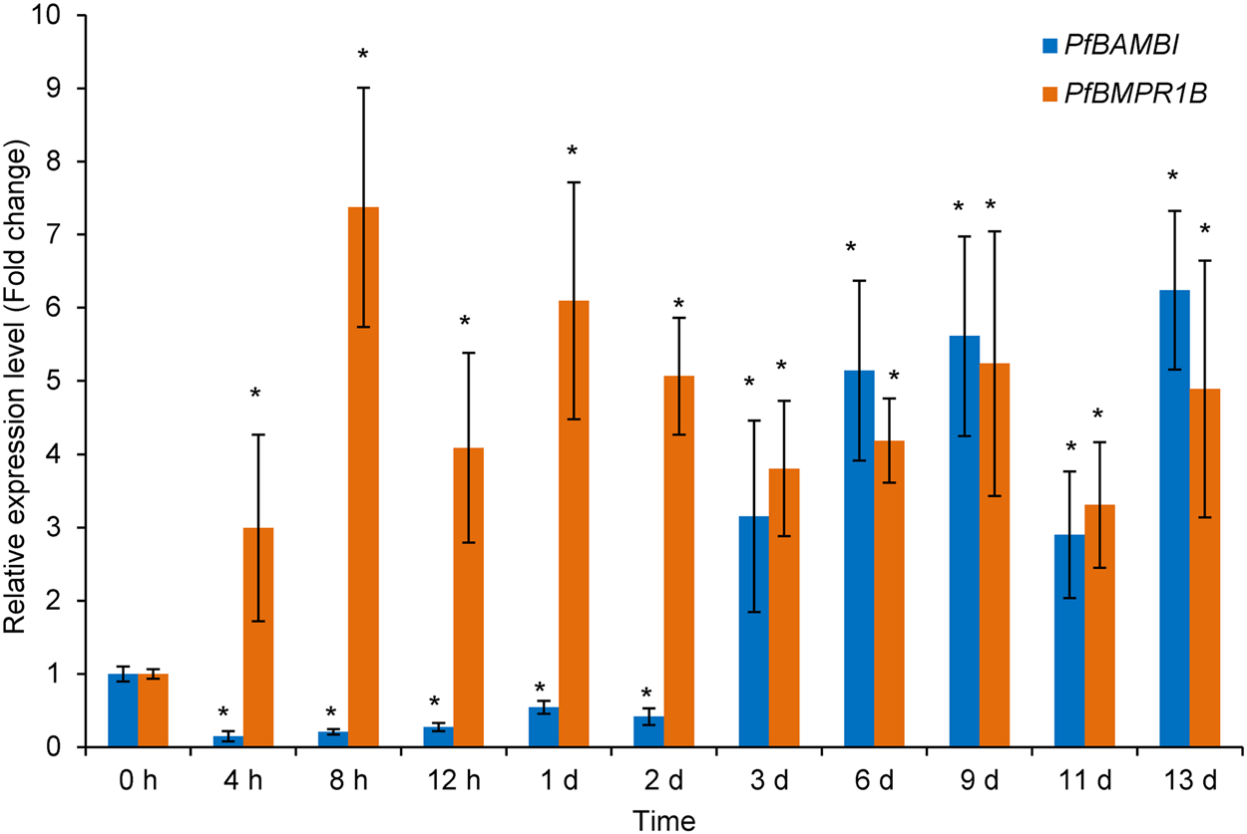
The relative expression levels of *PfBMPR1B* and *PfBAMBI* in the mantle of *Pinctada fucata* during shell notching. The expression levels of *PfBMPR1B* and *PfBAMBI* at 0 h are considered unnotched controls. *P < 0.05. Differences were assessed by one-way ANOVA.

As shown in Fig. 6, the relative expression levels of *PfBMPR1B* are 3.00-fold at 4 h, 7.37-fold at 8 h, 4.09-fold at 12 h, 6.10-fold at 1 d, 5.07-fold at 2 d, 3.81-fold at 3 d, 4.19-fold at 6 d, 5.24-fold at 9 d, 3.31-fold at 11 d and 4.89-fold at 13 d higher than at 0 h. The expression levels of *PfBMPR1B* show a rapid upward trend in the early stages of shell notching and a slight decrease in the later stages of shell notching, indicating that *PfBMPR1B* maintains a relatively high expression level after notching (*P* < 0.05). In contrast, the relative expression levels of *PfBAMBI* are 0.15-fold at 4 h, 0.21-fold at 8 h, 0.27-fold at 12 h, 0.54-fold at 1 d, and 0.42-fold at 2 d lower that at 0 h and 3.16-fold at 3 d, 5.14-fold at 6 d, 5.61-fold at 9 d, 2.90-fold at 11 d and 6.24-fold at 13 d higher than at 0 h. A significant downward trend in the expression levels of *PfBAMBI* during the early period and an upward trend during the late period of shell notching are observed (*P* < 0.05). The results indicate that *PfBMPR1B* and *PfBAMBI* may be competitively involved in shell regeneration in *P*. *fucata*.

### *PfBMPR1B* and *PfBAMBI* influence MP gene expression and shell ultrastructure

**Figure 7:**
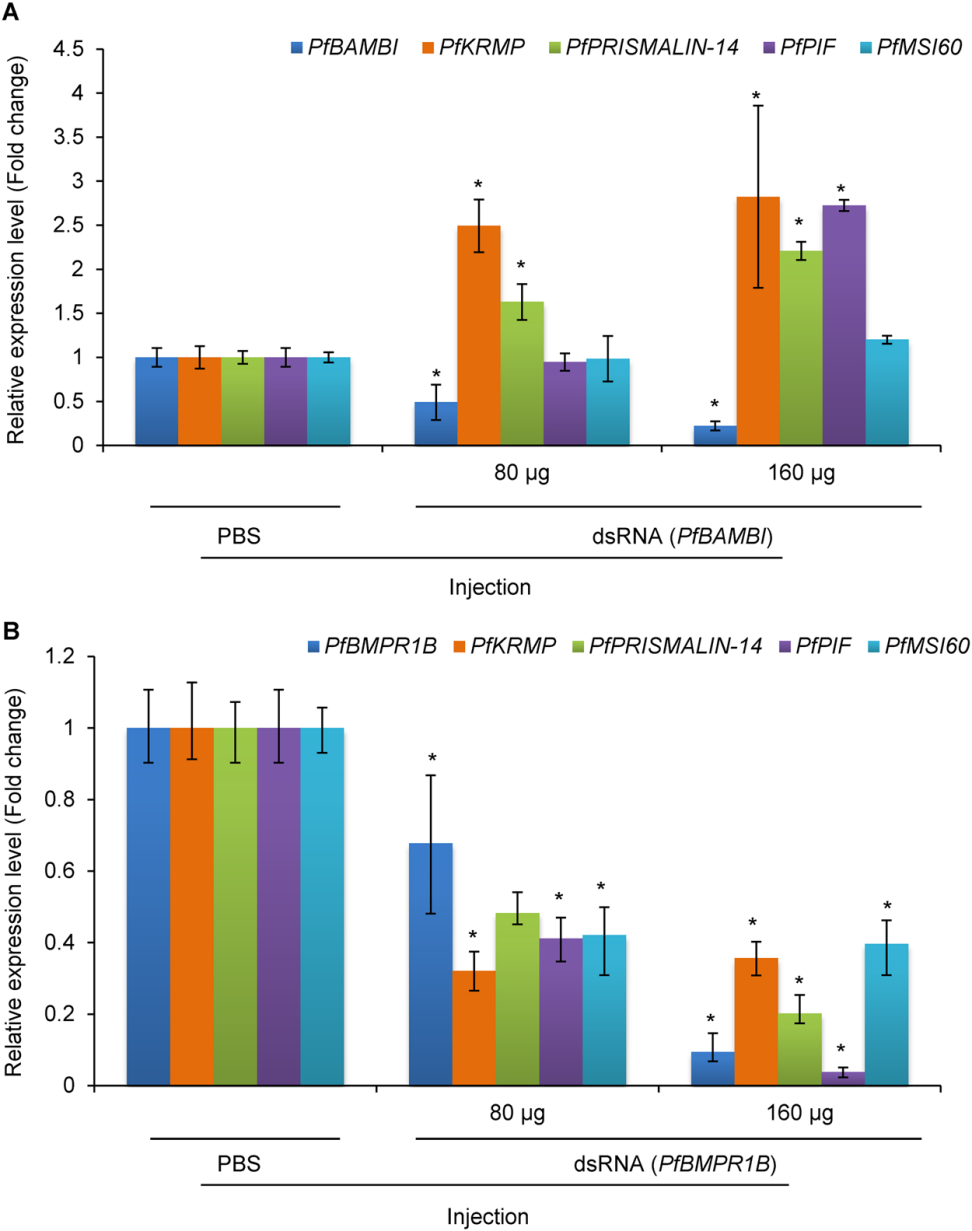
The relative expression levels of *PfBMPR1B*, *PfBAMBI* and shell matrix protein (MP) genes inhibited by RNA interference. (**A**) The expression of *PfBAMBI* and the MP genes *PfKRMP*, *PfPRISMALIN-14*, *PfPIF* and *PfMSI60* after injecting 80 and 160 μg of double-stranded RNA (dsRNA) of *PfBAMBI* for 6 d. (**B**) The expression of *PfBMPR1B* and the MP genes *PfKRMP*, *PfPRISMALIN-14*, *PfPIF* and *PfMSI60* after injecting 80 and 160 μg of double-stranded RNA (dsRNA) of *PfBMPR1B* for 6 d. PBS-injected groups were used as controls. *P < 0.05. Differences were assessed by one-way ANOVA.

Six days after injection, the relative expression levels of *PfBAMBI* were 0.49-fold (80 μg double strand RNA, dsRNA) and 0.22-fold (160 μg dsRNA) less than that in the control (Fig. 7A, *P* < 0.05). Corresponding to the decreases in the expression level of *PfBAMBI*, the expression levels of *PfKRMP* were significantly increased by 149.29% for the 80 μg and 182.37% for the 160 μg injection groups (*P* < 0.05), and the expression levels of *PfPRISMALIN-14* showed a significant increase of 62.88% for the 80 μg and 120.78% for the 160 μg injection group, respectively (*P* < 0.05). In addition, the expression levels of *PfPIF* and *PfMSI60* showed an increase of 172.46% and 20.06% for the 160 μg injection group, respectively (*P* < 0.05). The results show that the inhibition of *PfBAMBI* increases the expression levels of MP genes.

Compared with the control, the expression levels of *PfBMPR1B* decreased by 32.24% and 90.59% in the pearl oysters injected with 80 μg and 160 μg of dsRNA, respectively (Fig. 7B, *P* < 0.05). The expression levels of *PfKRMP*, *PfPRISMALIN-14*, *PfPIF* and *PfMSI60* decreased by 67.90%, 51.75%, 58.85% and 57.93% for the 80 μg injection and decrease by 64.35%, 79.38%, 96.21% and 60.38% for the 160 μg injection of *PfBMPR1B* dsRNA, respectively (Fig. 7B, *P* < 0.05), demonstrating that the expression levels of the MP genes are significantly inhibited by the depression of *PfBMPR1B*.

**Figure 8:**
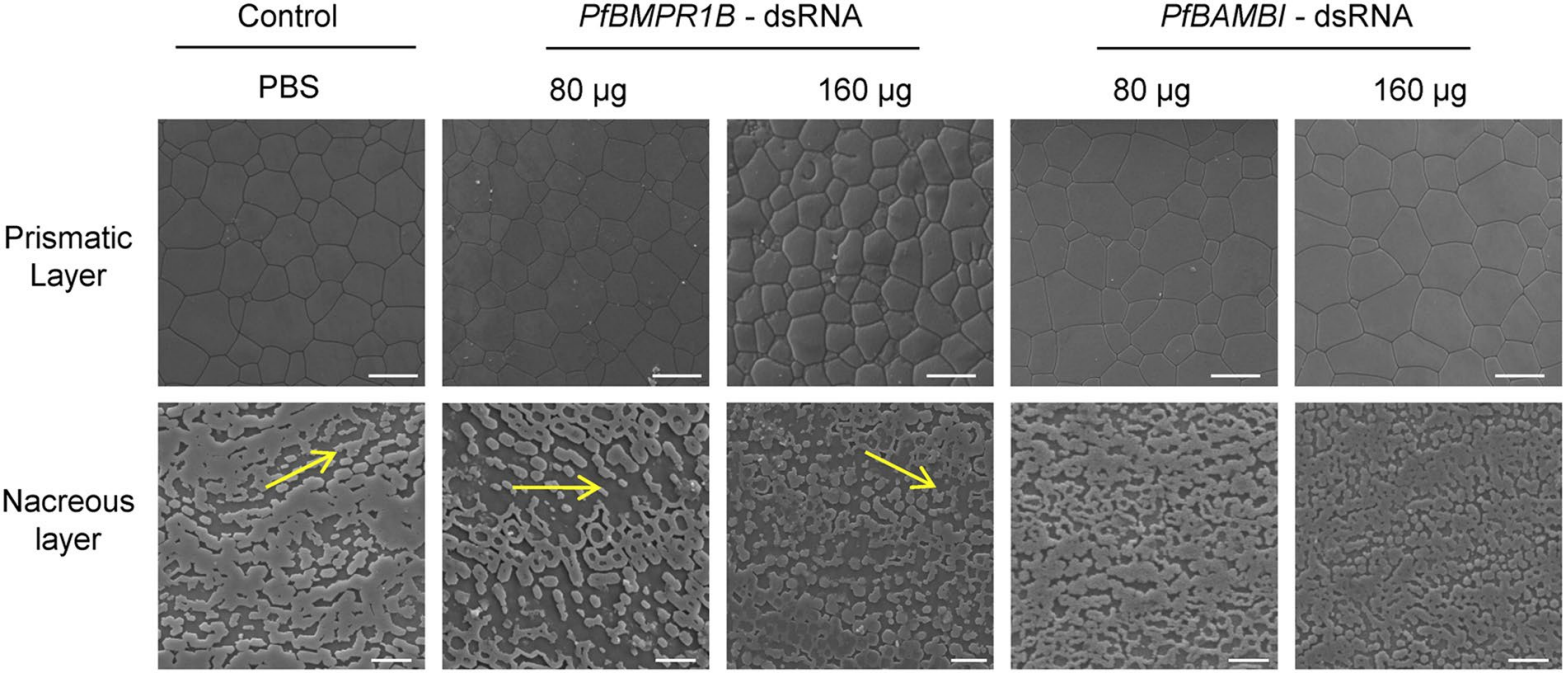
The scanning electron microscope (SEM) images of the ultrastructure of shell inner surface in *Pinctada fucata* influenced by RNA interference. Bar = 50 μm for the prismatic layer and Bar = 20 μm for the nacreous layer. The yellow arrows indicate the growth lines of aragonite crystals on the nacreous layer.

As shown in Fig. 8, the normal growth patterns of surface ultrastructure on the prismatic and nacreous layers are observed in the pearl oysters injected with phosphate buffer solution (PBS). The prismatic layer contains columnar calcite crystals and the nacreous layer contains aragonite crystals with hexagonal flat tablets. There are also clear growth lines on the nacreous layer (yellow arrows). For *PfBMPR1B*-dsRNA injection groups, the prismatic layer grew regularly under low dosage (80 μg), and abnormal characteristics with a few holes in the calcite crystals are observed under high dosage (160 μg). The changes in the nacreous layer are obvious, and incomplete aragonite crystals with holes are observed both in the low dosage (80 μg) and high dosage (160 μg) groups. For *PfBMPR1B*-dsRNA injection groups, the growth of the prismatic layer is regular both under the low dosage (80 μg) and high dosage (160 μg). However, the changes in the growth lines on the nacreous layer are obvious, and the holes are also observed in aragonite crystals, showing overgrowth of the aragonite crystals under the injection of *PfBMPR1B*-dsRNA.

### LDN193189 influences MP gene expression by disturbing Smad phosphorylation

**Figure 9:**
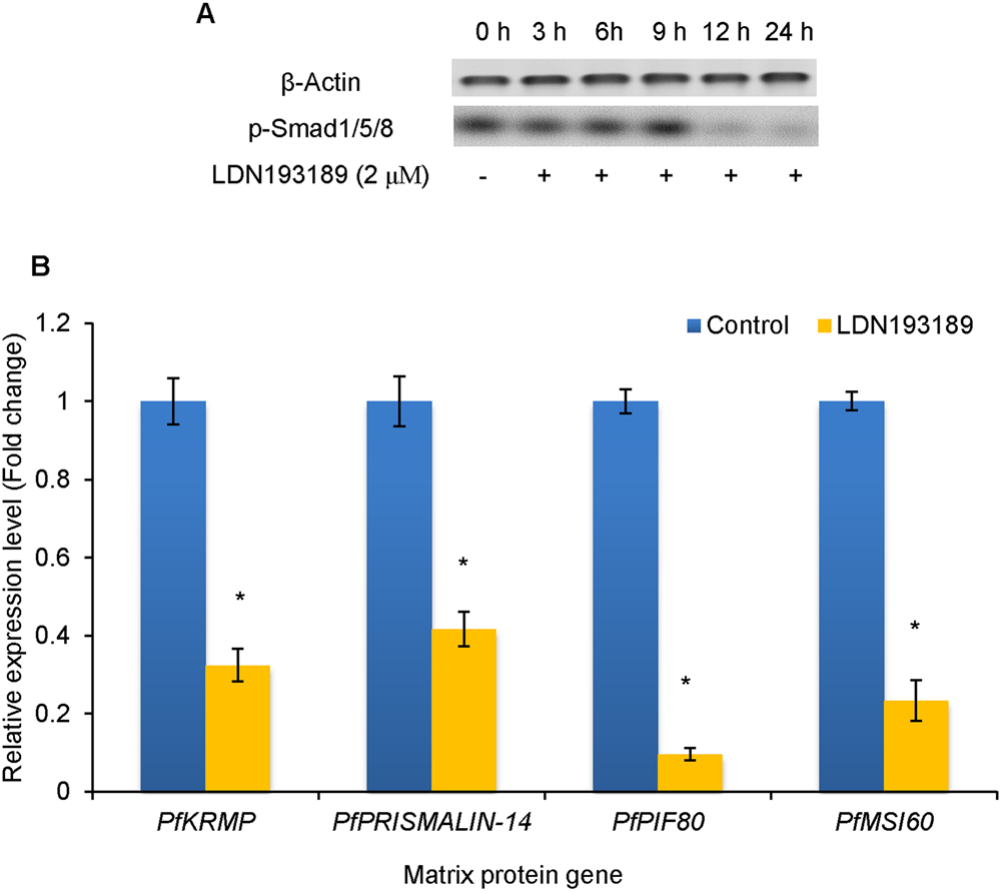
Effects of LDN193189 on the phosphorylation level of Smad1/5/8 and the expression level of matrix protein (MP) genes in mantle primary cells. (**A**) Changes in phosphorylated Smad1/5/8 in mantle cells detected by western blot. p-Smad1/5/8: Phosphorylated Smad1/5/8. β-Actin is used as control. (**B**) The relative expression levels of MP genes in the mantle cells exposed to LDN193189. The expression levels of MP genes in unexposed mantle cells were used as corresponding controls. *P < 0.05. Differences were assessed by one-way ANOVA.

The mantle cells in all groups grew healthy throughout the duration of exposure. Western blot analysis indicates that the phosphorylation levels of Smad1/5/8 significantly decreased in mantle cells exposed to 2 μM LDN193189 for 12 h and 24 h (Fig. 9A). When exposed to seawater with 2 μM LDN193189 for 12 h, the relative expression levels of *PfKRMP*, *PfPRISMALIN-14*, *PfPIF* and *PfMSI60* exhibit a decrease of 67.59%, 58.29%, 90.37% and 76.66%, respectively, compared with the control (Fig. 9B, *P* < 0.05). The results confirm that Bmpr1b is involved in the regulation of MP genes in mantle cells of *P*. *fucata*.

### LDN193189 influences shell germination during larval development

**Figure 10:**
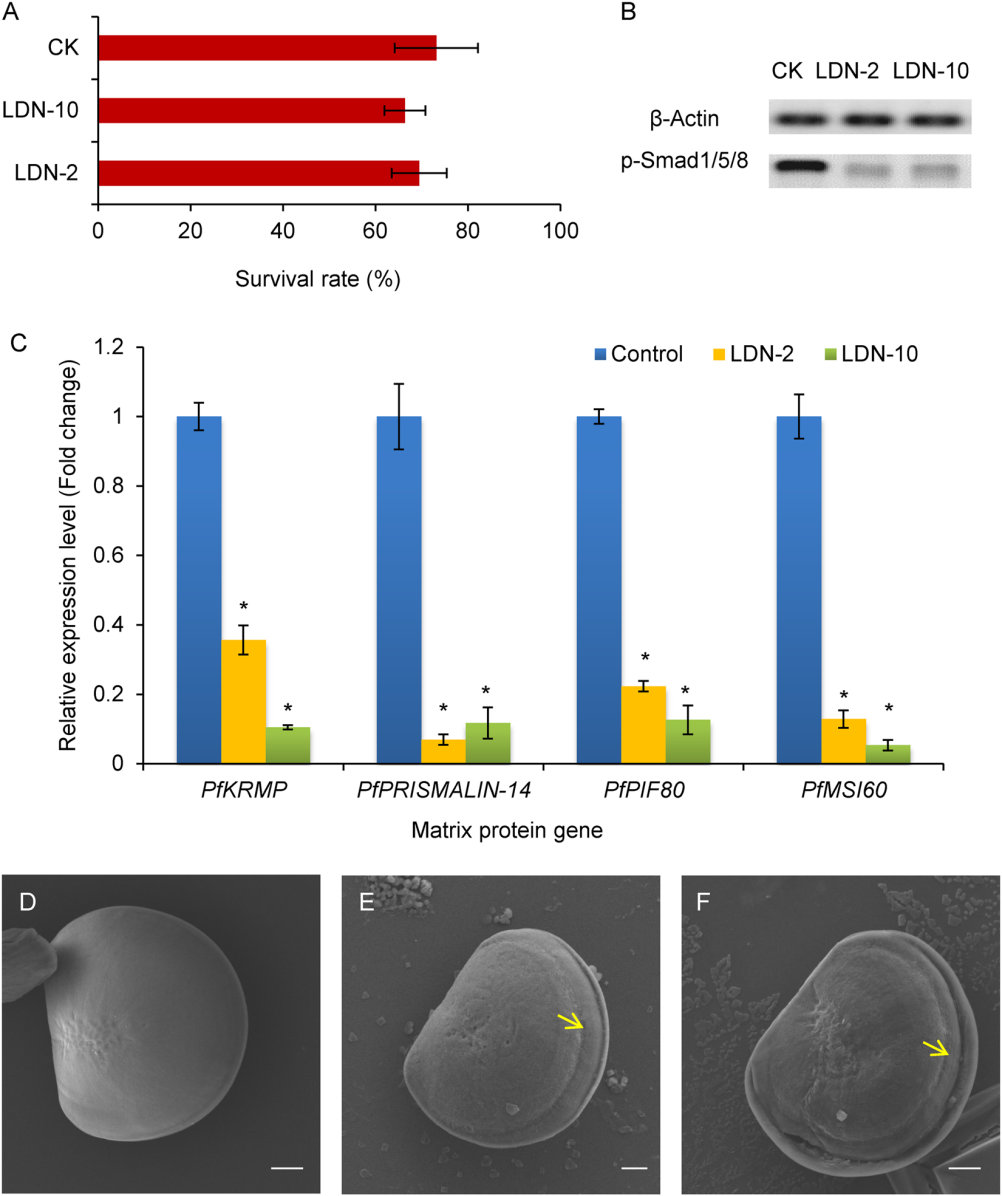
Effects of LDN193189 on shell germination during larval development in *Pinctada fucata*. (**A**) Survival rates of larvae exposed to different concentrations of LDN193189. CK: The Control larvae incubated in normal seawater. LDN-2: The larvae exposed to seawater with 2 μM of LDN193189 for 48 h. LDN-10: The larvae exposed to seawater with 10 μM of LDN193189 for 48 h. (**B**) Western blot analysis of the phosphorylation level of Smad1/5/8 in the larvae exposed to 2 μM and 10 μM LDN193189. p-Smad1/5/8: Phosphorylated Smad1/5/8. (**C**) The relative expression levels of MP genes in the larvae exposed to LDN193189. The expression levels of MP genes in unexposed larvae were used as corresponding controls. *P < 0.05. Differences were assessed by one-way ANOVA. (D-F) Scanning electron microscope (SEM) images of the shell surface of D-shaped larvae (Prodissoconch I) incubated in control (**D**), and 2 μM (**E**) and 10 μM (**F**) LDN193189. The yellow arrows indicate the wrinkled shell. Bar = 20 μm.

After exposure for 48 h, more than 95% of the larvae developed to the D-shaped stage, and 73.15%, 69.45% and 66.35% of the D-shaped larvae were alive in the control, 2 μM LDN193189 and 10 μM LDN193189 groups, respectively (Fig. 10A). There were no significant differences in the survival rates among these three groups (*P* < 0.05). For the surviving larvae, both 2 μM and 10 μM LDN193189 significantly decreased the phosphorylation level of Smad1/5/8 (Fig. 10B). When exposed to seawater with LDN193189 for 48 h, the relative expression levels of *PfKRMP*, *PfPRISMALIN-14*, *PfPIF* and *PfMSI60* exhibit a significant decrease of 64.36%, 93.05%, 77.87% and 87.12% for the concentration of 2 μM, and 89.53%, 88.29%, 87.37% and 94.65% for the concentration of 10 μM compared with the control (Fig. 10C, *P* < 0.05). LDN193189 exposure influences the morphology of the first original shell (prodissoconch I) at the D-shaped stage compared with the control (Fig. 10D,E and F). There is a deep dent in the margin of the D-shaped shell in the larvae exposed to 2 μM and 10 μM LDN193189, resulting in the formation of a wrinkled shell (Fig. 10E and F). The results indicate that the receptor Bmpr1b may be critical for shell germination during larval development in *P*. *fucata*.

## Discussion

The signaling pathways related to shell biomineralization have not been fully clarified, resulting in barriers for further understanding the molecular regulatory mechanism of mollusc biomineralization. In this study, two homologous genes of TGFβ/BMP signaling pathway receptors, *PfBAMBI* and *PfBMPR1B*, were cloned and identified from the pearl oyster *P*. *fucata*, and the potential roles in shell biomineralization were deeply investigated using multidisciplinary approaches. The obtained results here provide the first direct evidence that BMP receptors participate in mollusc biomineralization.

Consistent with the findings in vertebrates^20^, the amino acid sequence of *PfBMPR1B* contains a BD, TD, and kinase domain (KD), as well as an SP and GS domains (Fig. 1), which are the typical characteristics of BMP type I receptors. The GS domain, a highly-conserved region next to the KD in type I receptors in vertebrates, is essential for downstream Smad activation. Meanwhile, the amino acid sequence of *PfBAMBI* contains a BD, TD, KD and SP domains but lacks a GS domain (Fig. 1). These are the typical characteristics of BMP pseudoreceptors. According to these results, *PfBMPR1B* and *PfBAMBI* are proposed to be members of the TGFβ/BMP receptor families. Phylogenetic tree and Y2H analyses support this proposition (Figs 2 and 3), implying that the structures and functions of the receptors in the TGFβ/BMP signaling pathway are conserved. There are two kinds of BMP type I receptor genes (*BMPR1*) in vertebrates, classified as *BMPR1A* and *BMPR1B*. *BMPR1* expanded rapidly from protozoans to metazoans and evolved into these two subfamilies from the beginning of cephalochordate^32^. In *C*. *gigas* ^33^ and the sea urchin *Strongylocentrotu spurpuratus* ^34^, the related homologous genes have been named as *BMPR1*. Inconsistent with these findings, our study shows that the *BMPR1* homologous gene in *P*. *fucata* is similar to *BMPR1B*, and it was defined as *PfBMPR1B*. Further analyses for *BMPR1* using the genome of *C*. *gigas* ^33^, the gastropod snail *Lottia gigantean* ^35^ and the striped venus *Chamelea gallina* ^36^ draw the same conclusion (data not shown). Interestingly, the *BAMBI* homologous gene in *S*. *spurpuratus* was also not obtained from its genome. The authors predicted the following two reasons: *BAMBI* may have appeared after the emergence of echinoderms, or *BAMBI* may be lost in echinoderms^34^. Our results support the latter one because *BAMBI* has been found in various molluscs, including *C*. *gigas*, *L*. *anatina* and *P*. *fucata*.

The mantle is a well-known biomineralization-related tissue in shelled molluscs. Distributions of genes in the mantle can reflect the involvement of these genes in shell formation^37^. Generally, the genes expressed at the mantle edge are thought to be concerned with the formation of the prismatic layer and at the mantle pallial are thought to be concerned with the formation of the nacreous layer. The expression and location patterns (Figs 4 and 5) demonstrate the involvement of *PfBMPR1B* and *PfBAMBI* in shell biomineralization during prismatic and nacreous layer formation. This involvement is also confirmed by the different expression patterns of these genes during shell regeneration detected by shell notching (Fig. 6). Combined with the gene structures, we proposed that the differential expression might be a result of competitive regulation between *PfBMPR1B* and *PfBAMBI* to balance shell growth in notched pearl oysters. The rapid and high expression of *PfBMPR1B* can timely initiate shell biomineralization by stimulating the TGFβ/BMP signaling pathway to repair the damaged shells. In the meantime, the delayed expression of *PfBAMBI* may play the role of negative regulation in this signaling pathway, preventing excessive growth of the regenerated shells. The abnormal growth and overgrowth of the crystals on prismatic and nacreous layers were observed in RNAi experiments (Fig. 8). The proposition regarding the negative regulation of *BAMBI* has also been found in various species. For example, *BAMBI* negatively regulates TGFβ signaling during *Xenopus* embryogenesis^22^. *BAMBI* has a competing role with TGFβ during dentinogenesis in mouse MD10-A2 odontoblasts^38^. The absence of Bambi in chick wing generated a normal digit tip, indicating that it could be involved in terminal phalange homeostasis during chick wing formation^39^. In terms of the functional conservation of *PfBMPR1B* and *PfBAMBI*, the combined regulation of these genes on the TGFβ/BMP signaling pathway may control the homeostasis of shell biomineralization in *P*. *fucata*.

Understanding how *PfBMPR1B* and *PfBAMBI* regulate MP genes may be an effective way to uncover the role of TGFβ/BMP signaling pathway in shell biomineralization. The locations of *PfBMPR1B* and *PfBAMBI* in mantle tissue are coincident with that of some MPs, such as *MSI60*, which is located at the outer epithelia of the mantle edge and mantle pallial^40^, and *PRISMALIN-14*, which is located in the outer epithelia of the mantle edge^41^. This establishes a potential link between the receptors and MPs. MPs are key proteins that control shell formation in molluscs, but little is known about how these proteins are regulated. Knockdown of *PfBMPR1B* and *PfBAMBI in vivo* altered the expression levels of *PfKRMP*, *PfPRISMALIN-14*, *PfPIF* and *PfMSI60* (Fig. 7) and influenced surface ultrastructure of prismatic and nacreous layers (Fig. 8) in *P*. *fucata*. The selected MP genes in this study are specific for regulating shell formation. *PfKRMP* and *PfPRISMALIN-14* are proposed to be responsible for prismatic layer formation, and *PfPIF* and *PfMSI60* are proposed to be responsible for nacreous layer formation^37^. The down-regulation of *PfKRMP*, *PfPRISMALIN-14*, *PfPIF* and *PfMSI60* may lead to abnormal growth of CaCO_3_ crystals, which is similar to that observed in pearl oysters directly injected with dsRNA for corresponding MP genes^42, 43^. The up-regulation of *PfPIF* leads to changes in overgrowth of CaCO_3_ crystals. These may be the molecular mechanisms of the involvement of *PfBMPR1B* and *PfBAMBI* in shell biomineralization. Indeed, the types and amounts of MPs regulated by the TGFβ/BMP signaling pathway are not limited to the four types listed in our study, because shell biomineralization is a process that is controlled by multiple MPs. Although the specific mechanism is largely unknown, the study of the regulation of TGFβ/BMP receptors on MPs still has important biological significance.

Larva is an essential developmental stage for shell germination in molluscs, especially the D-shaped stage which starts from approximately 24 h after fertilization. The first original shell, prodissoconch I, forms during the early D-shaped stage. The subsequent prodissoconch II appears at the late D-shaped stage and umbonal stage^43^. LDN193189 inhibited the phosphorylation levels of Smad1/5/8 in exposed larvae, resulting in observable changes in the prodissoconch I shell morphology (Fig. 10). LDN193189 is a selective BMP signaling antagonist that inhibits the kinase activity of BMP type I receptors, including Bmpr1a, Bmpr1b and Acvr1. Using mouse as a model, LDN193189 inhibited the phosphorylation of Smad1, Smad5 and Smad8, leading to a reduction in ectopic ossification and functional impairment in mice^44^. LDN193189 ameliorated mineralization formation of human dental pulp cells by inhibiting BMP activation^45^. These investigations showed that inhibition of the BMP signaling pathway influenced bone and tooth biomineralization in vertebrates. Similar to these effects, a deep dent was observed on the shell margin at the prodissoconch I stage in the larvae exposed to LDN193189 (Fig. 10). Although our result cannot ensure which receptor was influenced by LDN193189, it shows that the TGFβ/BMP signaling pathway acts on shell germination of *P*. *fucata*. The involvement of the BMP signaling pathway in larval shell biomineralization was also observed in *L*. *anatine*, a brachiopod with phosphate biomineralization products^16^. The larval shell of shelled molluscs is formed by a shell gland, which is composed of a special kind of ectodermal cell in early embryos^46^. The inner part of these ectodermal cells is subsequently transformed into mantle epithelium of the larva. Studies on the mussel *M*. *galloprovincialis* and the European oyster *Ostrea edulis* ^47^ have shown that the organic matrix of the shell begins to be secreted by the shell gland during the late trochophore stage. Meanwhile, biomineralization products were detected at the inner side of the organic matrix at the early veliger stage. As a component of the organic matrix, MPs play important roles in shell germination^48^. The morphological changes in the prodissoconch I in the larvae of *P*. *fucata* may be caused by alterations of MPs, because *in vitro* study in mantle primary cells and *in vivo* study in larvae illustrate that LDN193189 decreases the expression levels of these genes (Figs 9 and 10). Interestingly, LDN193189 did not induced significant changes in the survival rate of *P*. *fucata* larvae. The reason for this phenomenon is largely unknown and needs further investigation. Although not conclusive, one piece of evidence implies that type I receptors, particularly *PfBMPR1B*, are critical for biomineralization during the shell germination stage.

**Figure 11:**
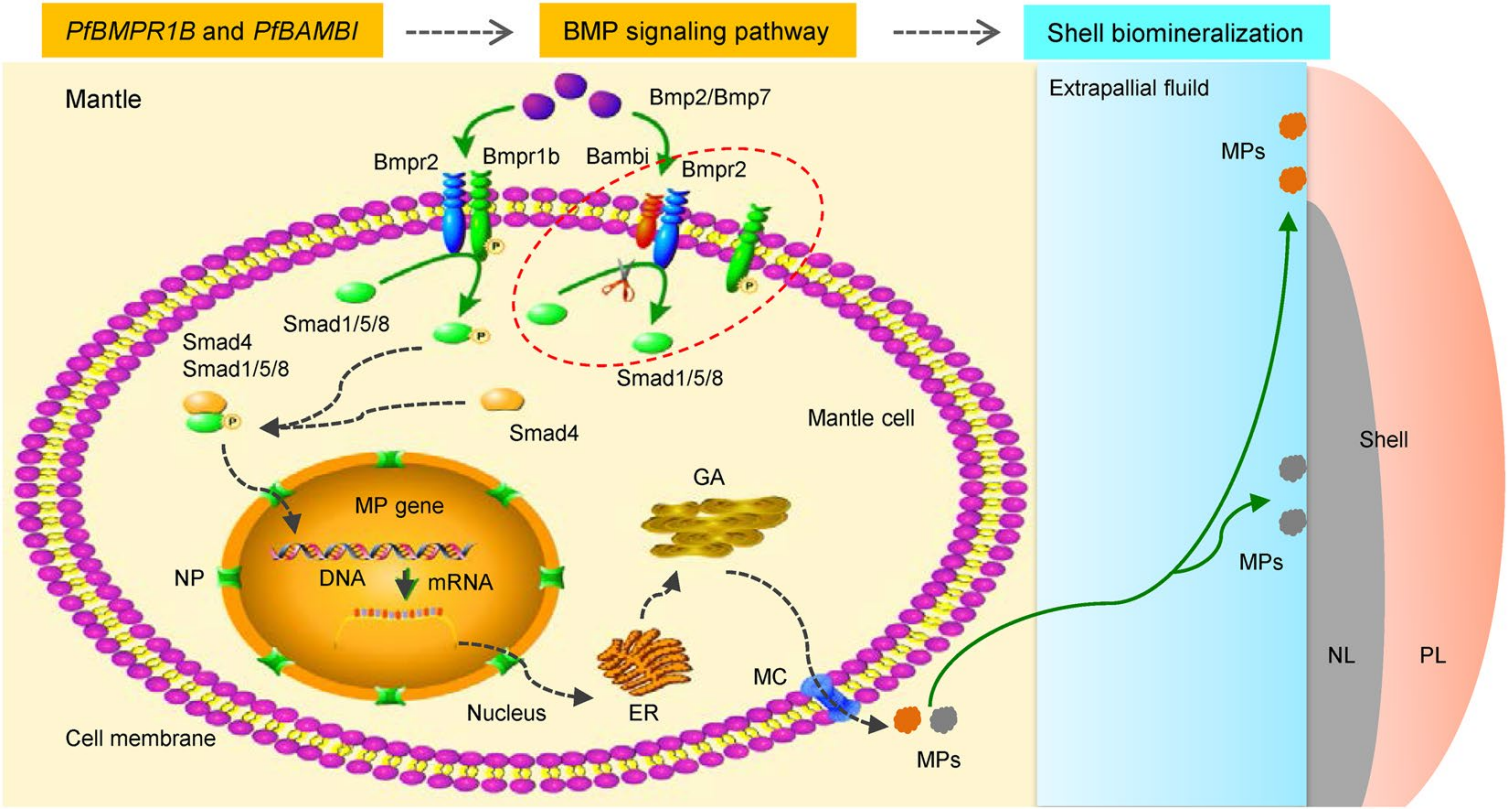
Schematic presentation of the mechanism for shell biomineralization mediated by the TGFβ/BMP signaling pathway in molluscs. In the mantle, the ligand Bmp2 or Bmp7 binds to the complex of type I receptor Bmpr1b and type II receptor Bmpr2. Bmpr1b is phosphorylated by the constitutively active Bmpr2 kinase and thereby recruits a cytoplasmic Smad1/5/8 (R-Smad), which is then phosphorylated by activated Bmpr1b. Smad1/5/8 may dissociate from the receptors to form a heteromeric complex with Smad4 (Co-Smad). The Smad complex is transported into the nucleus, where it regulates the expression of specific matrix protein (MP) genes alone or by binding certain transcription factors (either corepressors or coactivators). The MPs may be synthesized through conventional ways throughout the endoplasmic reticulum (ER) and Golgi apparatus (GA) and secreted out of the mantle cells. Finally, the MPs arrive at the mineralization site on the surface of the shell, and the MPs with different functions regulate the formation of the nacreous layer (NL) and prismatic layer (PL). Bambi is a negative regulator and competitively binds to the ligands and Bmpr2, which cannot trigger the phosphorylation of Smad1/5/8, inhibiting the signal transduction in this pathway. The green arrows indicate known pathways, and the gray dotted arrows indicate potential pathways. The red dotted circle indicates the interactions of Bambi, Bmpr2 and Bmpr1b. NP: Nuclear pore. MC: Membrane channel.

In conclusion, *PfBMPR1B* and *PfBAMBI* participate in the regulation of shell biomineralization and may be functional by regulating the expression of MP genes in a competitive manner. Our results therefore firmly support the hypothesis that TGFβ/BMP signaling pathway regulates mollusc biomineralization. Based on the findings from the investigations on ligands^12, 49^, receptors^14^ and Smads^11, 15^ in the TGFβ/BMP signaling pathway and the mechanism of MP-mediated biomineralization^5^ in shelled molluscs, we propose a hypothesis about the regulatory mechanism of the TGFβ/BMP signaling pathway in shell biomineralization (Fig. 11). Pearl is structurally similar to the nacreous layer of the shell in pearl oysters; thus, this hypothesis may also be suitable for understanding the mechanism of pearl formation. However, the precise mechanism by which the TGFβ/BMP signaling pathway regulates shell or pearl biomineralization should be deeply determined in the future studies.

## Methods

### Pearl oyster

Adult pearl oysters *Pinctada fucata* were obtained from the Marine Comprehensive Experimental Station, Chinese Academic of Sciences (Daya Bay, Shenzhen, China) in Feb 2015. All animals were transported immediately to our laboratory and maintained in an aerated aquarium with artificial seawater (Formula Grade A Reef Sea Salt, Formula, Japan) for two weeks prior to experimentation. Seawater conditions in the aquarium were 25.0 ± 0.3 °C, pH 8.1 ± 0.05, and salinity 33.0 ± 0.3, which were similar to the seawater in the sampling area.

### Gene cloning and sequence analysis

Total RNA was extracted from the mantle of adult *P*. *fucata* using TRIzol^**®**^ Reagent (Life Technologies, Carlsbad, CA, US) and further purified using a NucleoSpin RNA clean-up kit (Macherey-Nagel, Duren, Germany) following the manufacturer’s instructions. All RNA samples were quantified by measuring absorbance ratios using an ND-2000 spectrophotometer (NanoDrop Technologies, Wilmington, DE, USA) and electrophoresed on a 1.5% agarose gel to determine RNA integrity. A rapid-amplification of cDNA ends (RACE) method was employed to get the full length *PfBMPR1B* and *PfBAMBI* genes based on the expressed sequence tag (EST) of *PfBMPR1B* and *PfBAMBI* obtained from the mantle transcriptome of *P*. *fucata* ^31^. The complementary DNA library (cDNA) construction and PCR amplification for RACE were conducted with a SMARTer^®^RACE 5′/3′ Kit (TaKaRa, Tokyo, Japan) using the primers are listed in Supplementary Table 2.

The ORF, amino acid sequence, conserved domain, signal peptide and sequence characteristics of *PfBMPR1B* and *PfBAMBI* were analyzed using the ORF finder tool (http://www.ncbi.nlm.nih.gov/projects/gorf/), CBS Prediction Servers (http://www.cbs.dtu.dk/services/), ExPASY (http://www.expasy.org/tools/), SMART (http://smart.embl-heidelberg.de/) and ClustalX (http://www.clustal.org/clustal2/) programs. Phylogenetic tree was conducted using MEGA4.0 software (http://www.megasoftware.net/mega4/mega41.html). Several species that are not genetically related to molluscs, including fishes, rodents, chiropterans, primates, amphibians, birds, artiodactyls and reptiles, were served as the outgroups (see details in Fig. 2). Homologous sequences for Bmpr1a, Bmpr1b and Bambi from the outgroup species were selected to construct phylogenetic tree. These sequences were close enough for meaningful comparisons to the Bmpr1a, Bmpr1b and Bambi sequences in *P*. *fucata*. The phylogenetic tree was then divided artificially into subgroups according to the relationships of these homologous sequences and labeled with different colors.

### Gene expression analysis

To analyze the expression patterns of *PfBMPR1B* and *PfBAMBI* in different tissues, the mantle edge, mantle pallial, adductor, gonad, gill, foot and viscus of *P*. *fucata* were excised from ten individuals and mixed together as a biological replicate. Total RNA extraction, purification and quantification for three replicates were conducted using the method described above. The cDNA was synthesized using a PrimeScript™ RT Reagent Kit (Takara, Tokyo, Japan) according to the manufacturer’s instructions. The reverse transcription quantitative real-time PCR (RT-qPCR) was performed with 1 μL of cDNA, 0.4 μM of each primer (Supplementary Table 2) and 2 × SYBR Green Master Mix from a SYBR® *Premix Ex Taq*™ II kit (Takara, Tokyo, Japan) to obtain a total volume of 20 μL. The PCR reaction was run as follows: 1 cycle of 95 °C for 30 s; 40 cycles of 95 °C for 5 s, 60 °C for 30 s; and 1 cycle of 95 °C for 15 s, 60 °C for 1 min and 95 °C for 15 s. The fluorescent products were detected using a StepOnePlus™ Real-Time PCR system (Applied Biosystems, Foster, CA, US). *PfACTIN* (GenBank accession: AB252571.1) was used as the internal control and the 2^−ΔΔCT^ method^50^ was used to analyze the relative expression levels of *PfBMPR1B* and *PfBAMBI*.

### Shell notching

To detect the responses of *PfBMPR1B* and *PfBAMBI* to the shell repair process in *P*. *fucata*, a shell notching experiment was conducted following a previously reported method^51^ with minor modifications. Biological replicates were conducted in three separate aquariums. After notching for 4 h, 8 h, 12 h, 1 d, 2 d, 3 d, 6 d, 9 d, 11 d, and 13 d, the mantles from five individuals in an aquarium were sampled and mixed together as a biological replicate. The mantle from five unnotched individuals in an aquarium at each time point were also collected and considered a corresponding control. The total RNA from three replicates at each time point was extracted, purified and quantified. The relative expression levels of *PfBMPR1B* and *PfBAMBI* in these samples were analyzed using the RT-qPCR method.

### *In situ* hybridization

To further verify the accurate expression locations of *PfBMPR1B* and *PfBAMBI* in the mantle, *in situ* hybridization (ISH) was performed on frozen sections of the mantle. Mantle tissues were excised from adults and washed immediately with 0.1 M PBS. After being cut into little pieces, the tissues were immersed in 4% (w/v) paraformaldehyde solution, fixed overnight and moved into 20% (w/v) sucrose for another night to dehydrate the samples. A Leica frozen slicer CM1900 (Leica, Wetzlar, German) was used to obtain 10 μm frozen sections. The sense and anti-sense probes were synthesized by amplifying the ORF sequences of *PfBMPR1B* and *PfBAMBI* using the specific primers (Supplementary Table 2). The products were subcloned into the vector pGEM-T-Easy (Promega, USA) and then labeled with digoxigenin (DIG) using a DIG RNA Labeling (SP6/T7) Kit (Roche, Basel, Switzerland) following the manufacturer’s instructions. The sense probe was used as a control. An Enhanced Sensitive ISH Detection Kit II (Boster, Wuhan, China) was used to perform the hybridization according to the protocol described by Kong *et al*.^52^. The hybridization temperature was set at 50 °C to avoid false positive signals. Finally, the hybridization products were washed with 0.5 M TBS. Images were captured by a Mono Cooled Digital Camera Head (DS-Qi2, Nikon, Tokyo, Japan) and photographed with NIS Elements Version 3.3 software (MQS33000, Nikon, Tokyo, Japan).

### Yeast two-hybrid system

To clarify the interactions among the receptors, ligand and signaling transmitters of the TGF-β/BMP signaling pathway in *P*. *fucata*, Y2H screen was performed using a Matchmaker GAL4 Two-Hybrid System (Clontech, Tokyo, Japan) following the manufacturer’s instructions. Briefly, the plasmids were constructed by subcloning the ORF of *PfBMPR1B* and *PfSMAD4* into an EcoRI/BamHI digested pGADT7 vector (AD), the ORF of *PfSMAD1/5/8* and *PfBMP2* into a CalI/BamHI digested pGADT7 vector, and the ORF of *PfBAMBI* and *PfBMPR1B* into an EcoRI/PstI digested pGBKT7 vector (BD) using the primers incorporated with corresponding restriction enzyme cutting sites (Supplementary Table 2). The fused plasmids were transformed separately into the yeast strain AH109. The transformed yeasts were then spotted on SD medium to detect the toxicity of the fused plasmids. Meanwhile, the transformed yeasts were also spotted on SD-Trp-Leu medium to ensure the effects of self-activation. After verifying the efficiency of the Y2H system, the prey and bait plasmids, including AD and BD-*PfBAMBI*, AD and BD-*PfBMPR1B*, BD and AD-*PfBMP2*, BD and AD-*PfSMAD1/5/8*, BD and AD-*PfSMAD4*, BD and AD-*PfBMPR1B*, BD-*PfBAMBI* and AD-*PfBMP2*, BD-*PfBAMBI* and AD-*PfSMAD1/5/8*, BD-*PfBAMBI* and AD-*PfBMPR1B*, BD-*PfBMPR1B* and AD-*PfBMP2*, BD-*PfBMPR1B* and AD-*PfSMAD1/5/8*, BD-*PfBMPR1B* and AD-*PfSMAD4*, BD-*PfBAMBI* and AD-*PfSMAD4*, were cotransformed into the yeast strain AH109 using lithium acetate method following the manufacturer’s instructions. The cotransformed yeast was spotted on SD-Trp-Leu and SD-Trp-Leu-His-Ade/X-α-gal mediums. Cotransformations of AD-T and BD–p53, AD-T and BD-Lam, AD and BD were used as positive, negative and empty plasmid controls. The AH109 yeasts were finally incubated at 30 °C for 72~96 h, and the yeast plaques were recorded by a digital camera (EOS 5D, Canon, Tokyo, Japan).

### RNA interference

To reveal the effects of *PfBMPR1B* and *PfBAMBI* on the expression of MP genes and shell ultrastructure, RNA interference (RNAi) was performed as described by Fang *et al*. (2012)^43^ with minor modifications. The linear DNA sequences were amplified using the specific primers listed in Supplementary Table 2. After diluting the dsRNA with 0.1 M PBS to final concentrations of 80 and 160 μg/100 μL, 80 and 160 μg of dsRNA probes were injected into the adductor of *P*. *fucata*. Instead of the probe, the control group was injected with the same volume of 0.1 M PBS solution. The treated pearl oysters were incubated for 6 d.

RT-qPCR was conducted to investigate the effects of RNAi on the expression of the MP genes *PfKRMP*, *PfPRISMALIN-14*, *PfPIF* and *PfMSI60* using the specific primers (Supplementary Table 2). The shells were also collected, cut into small pieces, washed with sterilized water and air-dried. The shells collected from 10 individuals were mixed as a biological replicate, and shells collected from PBS-injected individuals were used as a control. Thirty samples for the nacreous and prismatic layers of the shell inner surface (near the nacre-prism transition region) from three replicates were observed by scanning electron microscope (SEM, FEI Quanta 200, Netherlands) following a described protocol^53^. The shell samples were sputter-coated with a nanoporous film of gold for 60 seconds.

### Mantle cell exposure

To study the effects of the BMP type I receptor on the expression of the MP genes *in vitro*, the primary cells of mantle tissue were cultured and treated with the receptor inhibitor LDN193189 (Sigma-Aldrich). The mantle pallial was excised from the edge of mantle in 30 adult pearl oysters and the primary cells of the mantle tissue were acquired according to the method described by Gong *et al*.^54^. The mantle cells were incubated in medium with 2 μM LDN193189 for 3, 6, 9, 12 and 24 h to access the effects of exposure time. The mantle cells were collected and lyzed with TNE buffer supplemented with 1% protease inhibitor cocktail and separated by sodium dodecyl sulfate polyacrylamide gel electropheresis (SDS-PAGE). Western blot analysis was conducted according to the protocol described by Xiang *et al*.^55^. The primary antibody used for the western blot was rabbit monoclonal anti-phospho-SMAD1/5/8 at 1:600 dilution (#9516S, Cell Signaling, MA). The secondary antibody was HRP-conjugated goat anti-rabbit (Calbiochem) at 1:1000 dilution. β-actin was used as the loading control^56^.

For LDN193189 exposure, the mantle cells were incubated in medium with 2 and 10 μM LDN193189 in 6-well cluster dishes. After exposure for 12 h, the mantle cells were centrifuged at 500 × g for 5 min, washed with 1 × PBS and centrifuged again at 500 × g for 5 min. The total RNA was extracted from the collected cells, and RT-qPCR was performed, following the protocol mentioned above, to detect the relative expression levels of the MP genes *PfKRMP*, *PfPRISMALIN-14*, *PfPIF* and *PfMSI60*. Mantle cells incubated in normal medium were used as the control.

### Larvae exposure

To verify the role of the BMP type I receptor in shell germination, LDN193189 was used to disturb Smad phosphorylation at the early developmental stage of *P*. *fucata*. Artificial insemination was employed to obtain oosperm from sexually mature pearl oysters according to a protocol described by Liu *et al*.^57^. The experiments were conducted in 6-well cluster dishes with 5 mL of filtrated seawater. A total of 2000 fertilized eggs were exposed to seawater with 2 or 10 μM LDN193189 in each dish for 12 h, transferred into a 1 L breaker with 900 mL of filtrated seawater and exposed for an additional 36 h until they developed into the D-shaped stage. The same amount of larvae incubated in seawater without the receptor inhibitor was used as a control. Three biological replicates were used for each treatment, and each replicate contained three separate incubations. One-third of the exposure seawater was replaced daily with seawater containing an equal amount of LDN193189. During larval development, the embryo, trochophore and D-shaped stage were collected and observed by microscopy. The survival numbers and corresponding ratios were recorded.

Western blot analysis was conducted to detect the phosphorylation level of Smad1/5/8 in the larvae. RT-qPCR was performed, following the protocol mentioned above, to detect the relative expression levels of the MP genes *PfKRMP*, *PfPRISMALIN-14*, *PfPIF* and *PfMSI60*. The collected larvae were washed with sterilized seawater for three times, fixed with 4% paraformaldehyde (PFA) for 24 h, dehydrated by an ethanol gradient and air-dried. Subsequently, larvae were then spread on a cover glass, and SEM was used to observe detailed morphological changes, especially for the shell at the D-shaped stage.

### Statistical analysis

All the statistical analyses were performed using SPSS version 18.0 for Windows (SPSS Inc. Chicago, IL, USA). The values derived from three replicates were analyzed by one-way ANOVA to identify differences between groups at the 0.05 significant levels and are presented as the means ± standard deviation. All the figures were drawn using SigmaPlot version 12.5 (Systat Software, San Jose, CA, USA) and Excel (version 2010 for Windows).

### Data accessibility

Nucleotide sequences of *PfBMPR1B* and *PfBAMBI* are available in the GenBank database under the accession numbers KF280238.1 and KF280237.1, respectively.

## Electronic supplementary material

Supplementary Information
