# Supplementary material for: The receptor genes PfBMPR1B and PfBAMBI are involved in regulating shell biomineralization in the pearl oyster Pinctada fucata
Source: Sci Rep. 2017 Aug 23;7:9219. doi: 10.1038/s41598-017-10011-y (PMC5569090; doi:10.1038/s41598-017-10011-y)
Supplement: Supplementary file 1 — Supplementary Information [file 41598_2017_10011_MOESM1_ESM.pdf]

# The receptor genes *PfBMPR1B* and *PfBAMBI* are involved in regulating shell biomineralization in the pearl oyster *Pinctada fucata*

Shiguo Li[1,2§], Yangjia Liu[1§], Jingliang Huang[1], Aibin Zhan[2], Liping Xie[1]* & Rongqing Zhang[1,3]*

[1] Institute of Marine Biotechnology, School of Life Sciences, Tsinghua University, Beijing 100084, China. [2] Research Center for Eco-Environmental Sciences, Chinese Academy of Sciences, Beijing 100085, China. [3] Department of Biotechnology and Biomedicine, Yangtze Delta Region Institute of Tsinghua University

[§] These authors contributed equally to this work and should be considered co-first authors

* Corresponding aouthors:

Dr. Rongqing Zhang

rqzhanglab@tsinghua.edu.cn

Dr. Liping Xie

lpxie@tsinghua.edu.cn

## Supplementary Tables

**Supplementary Table 1**. Toxicity test of recombinant plasmids on the transformed yeast AH109. After incubation for 15 h, the $OD_{600}$ absorbance value of the AH109 yeast was measured. AD: pGADT7. BD: pGBKT7.

| Plasmid | $OD_{600}$ | Toxicity |
|---|---|---|
| AD | 1.225 | No |
| BD | 1.080 | No |
| AD-BMPR1B | 1.366 | No |
| AD-BMP2 | 0.901 | No |
| AD-SMAD1/5/8 | 0.877 | No |
| AD-SMAD4 | 0.809 | No |
| BD-BAMBI | 1.034 | No |
| BD-BMPR1B | 1.260 | No |

**Supplementary Table 2.** Primers used in the present study. RACE: Rapid amplification of cDNA ends. RT-qPCR: Reverse transcription real-time quantitative PCR. ISH: *In situ* hybridization. Y2H: Yeast two-hybrid system.

| **Primer name** | **Sequence (5'-3')** | **Application** |
|---|---|---|
| UPM: | CTAATACGACTCACTATAGGGCAAGCAGTGGTAT<br>CAACGCAGAGT | |
| NUP | AAGCAGTGGTAACAACGCAGAGT | |
| BMPR1B-GSP1 | TACAGTAATCCTCGTCATCACAGCAC | RACE |
| BMPR1B-NGSP1 | GAAGTTGTTGTATTGCTGATCTGGGATTAGC | |
| BAMBI-GSP1 | GCAATAGGGCCTCCATAAACTGTCAACC | |
| BAMBI-NGSP1 | TCCATAAACTGTCAACCCCCTCGTCCC | |
| RT-PfACTIN-F | TACCGCCGCGTCATCATCAT | |
| RT-PfACTIN-R | TGCCTCGGGACATCTGAACC | |
| RT-PfBAMBI-F | ACTGTACAAGGAAGGCGTGTCAC | |
| RT-PfBAMBI-R | GAGCTGGCATTTGTTTGGACGTG | |
| RT-PfBMPR1B-F | GCACATAACACACGGCAAGGAAC | |
| RT-PfBMPR1B-R | TCTGCTTGTCGGTAGGCTTCAAA | |
| RT-KRMP-F | GAATGAAGTTCGCCGCTGTT | RT-qPCR |
| RT-KRMP-R | TTCCAATCCCARGGRTGACA | |
| RT-PRISMALIN14-F | AAAGAAATACTTAACTGGTGCTA | |
| RT-PRISMALIN14-R | CATGAGCAGCCCGGGTC | |
| RT-PIF-F | TGCTGCCATCACGTGAGTATG | |
| RT-PIF-R | GACTTCCCTTTCTCACACTTCCA | |
| RT-MSI60-F | GAACAATGACTGGAATGACA | |
| RT-MSI60-R | GGAAAGGTATCCAATAACAAC | |
| ISH-BAMBI-F | GAGAAGAGGTGCTACTGC | |
| ISH-BAMBI-R | GCTGGATTCTGCTTCTCG | ISH |
| ISH-BMPR1B-F | GAGTGCTGTGATGACGAGGATTAC | |
| ISH-BMPR1B-R | TTGATTCACTTATATATCGCACTGC | |
| RNAi-BAMBI-F | GCGTAATACGACTCACTATAGGGAGATGCGATGT<br>TGTAAAGAGGA | |
| RNAi-BAMBI-R | GCGTAATACGACTCACTATAGGGAGATTTCACGA<br>CGGATTTGTAT | |
| RNAi-BMPR1B-F | GCGTAATACGACTCACTATAGGGAGACTGGCGAC<br>TCAATGTCTCAA | RNAi |
| RNAi-BMPR1B-R | GCGTAATACGACTCACTATAGGGAGACTGGCATT<br>GGTGTTGTTGAC | |
| BAMBI-EcoRI | CGGAATTCATGGAGGCCCTATTGCTTC | |
| BAMBI-PstI | AACTGCAGCTATACAGAAGCCACCAAGTC | Y2H |
| BMPR1B-EcoRI | CGGAATTCATGGCAGACCTCTGCTGG | |
| BMPR1B-PstI | AACTGCAGCTAGCTTTCTCCGGGTTTTATGAC | |

| Primer name | Sequence (5'-3') | Application |
|---|---|---|
| BMPR1B-BamHI | CGGGATCCCTAGCTTTCTCCGGGTTTTATGAC | |
| SMAD1/5/8-ClaI | CCATCGATATGAGTTCACCCATCTCC | |
| SMAD1/5/8-BamHI | CGGGATCCTCATGATACAGATGAAATTGGG | |
| BMP2-ClaI | CCATCGATATGATTTACGGATTTGGACATTACC | |
| BMP2-BamHI | CGGGATCCCTACCGACATCCGCATCC | |
| SMAD4-EcoRI | CGGAATTCATGTTTCGGTCTAAAAGATCTACCCTC | |
| SMAD4-BamHI | CGGGATCCTCACCTGTGGACGTTCAATAAAATTTC | |

## Supplementary Figures

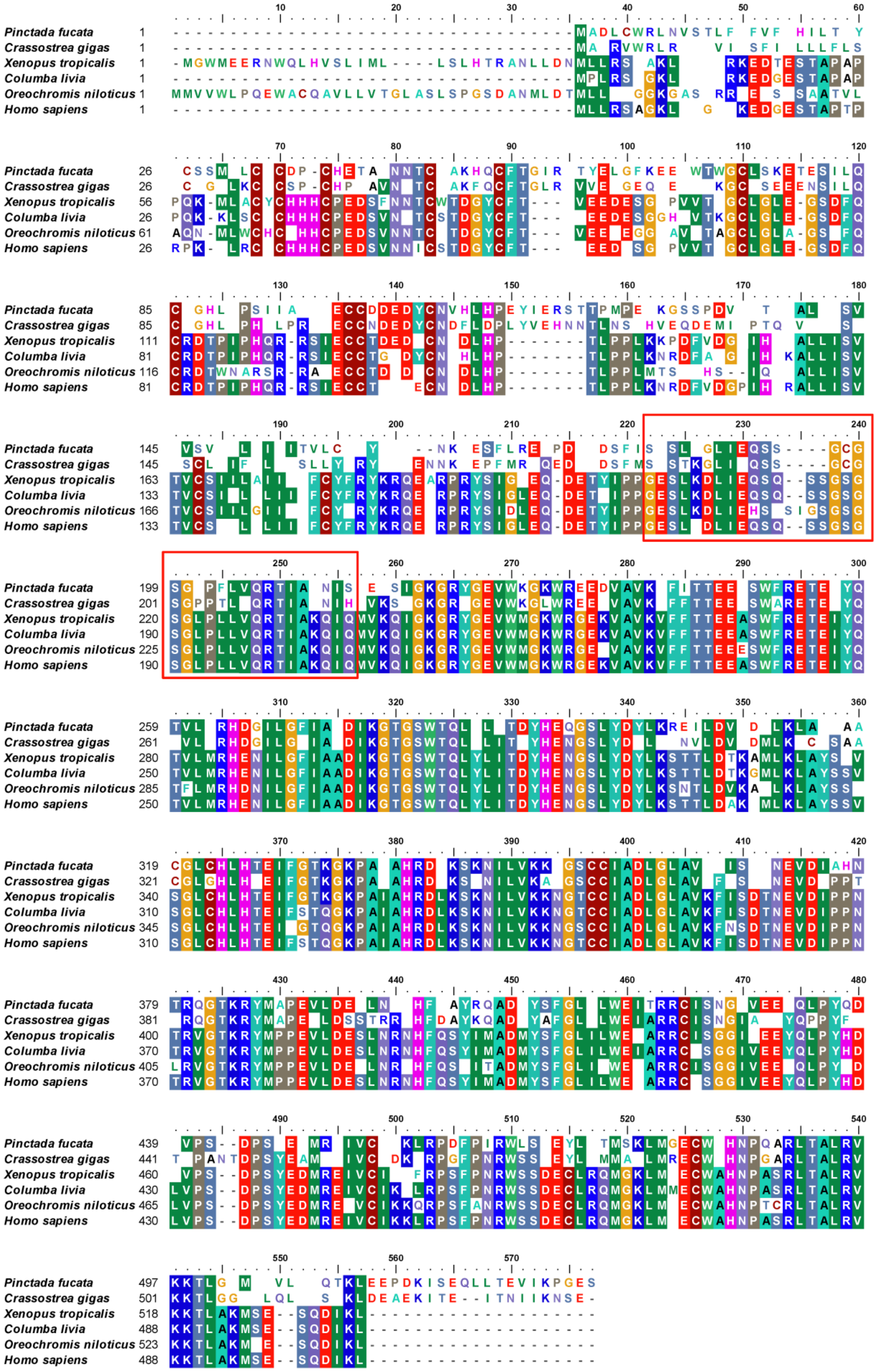

**Supplementary Figure 1.** Alignment of amino acid sequences for Bmpr1b homologues in six species. The GenBank accession numbers of different species are listed as follows: *Pinctada fucata* (AGW51569.1), *Crassostrea gigas* (CAE11917.1), *Xenopus tropicalis* (NP_001072633.1), *Columba livia* (XP_005498077.1), *Oreochromis niloticus* (XP_003453955.2) and *Homo sapiens* (AAH47773.1). The red boxes indicate the conserved glycine/serine-rich domain (GS) of these species.

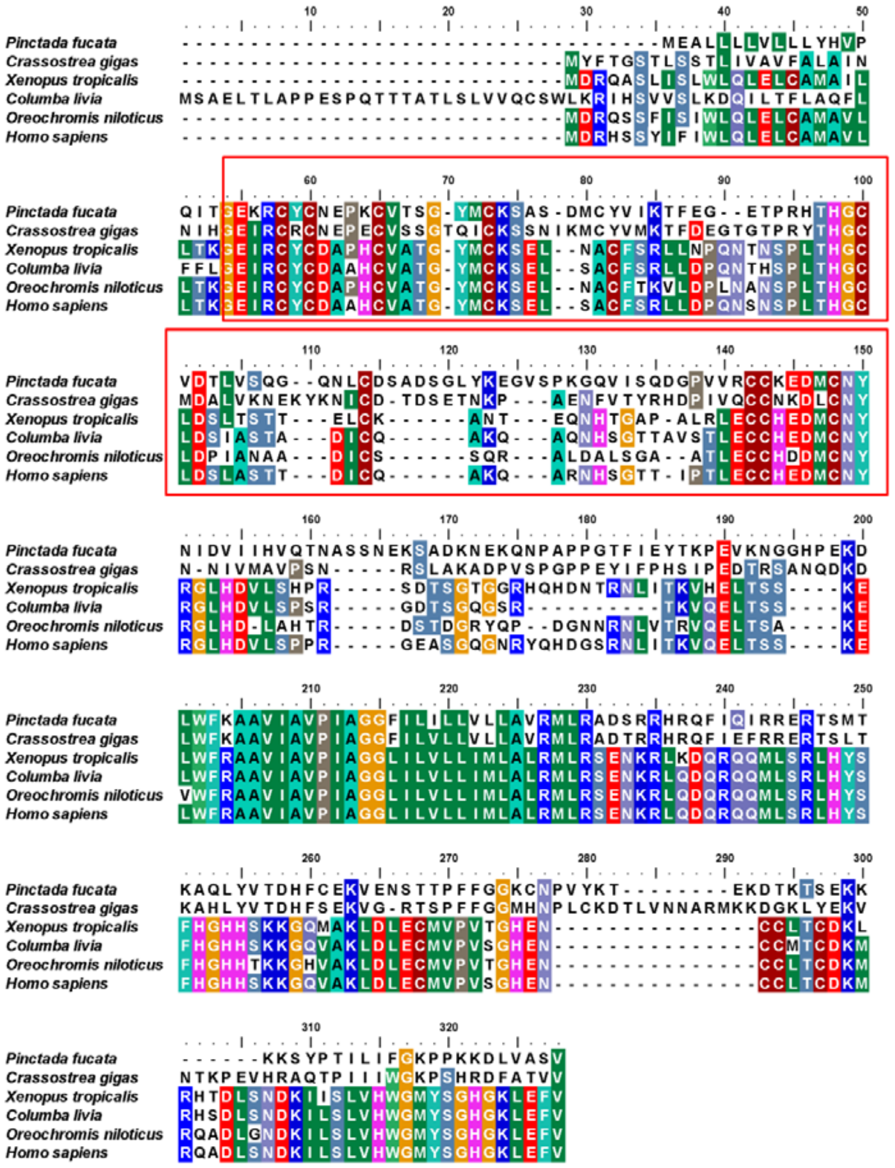

**Supplementary Figure 2.** Alignment of amino acid sequences for Bambi homologues in six species. The GenBank accession numbers of different species are listed as follows: *Pinctada fucata* (AGW51569.1), *Crassostrea gigas* (XP_011449669.1), *Xenopus tropicalis* (CAJ81778.1), *Columba livia* (XP_005504247), *Oreochromis niloticus* (XP_003450291.1) and *Homo sapiens* (NP_036474.1). The red boxes indicate the conserved Bambi domain of these species.

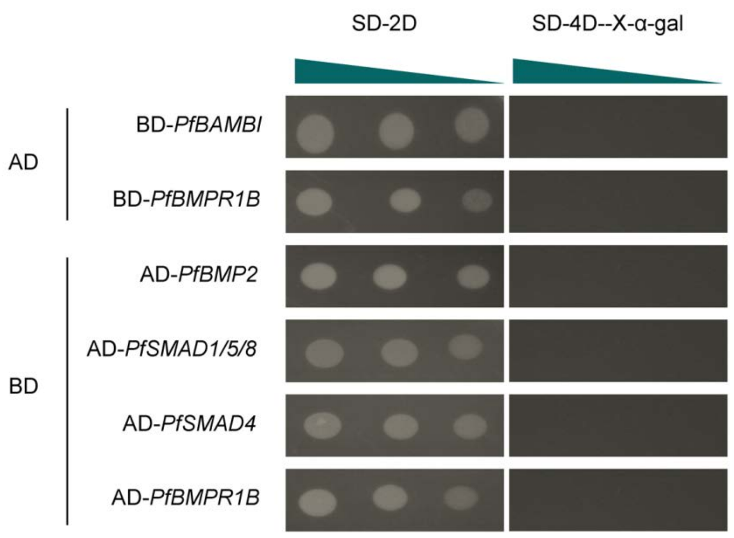


**Supplementary Figure 3.** Detection of the self-activation of recombined plasmids on reporter genes in transformed AH109. AD: pGADT7. BD: pGBKT7. SD-2D: SD-Leu-Trp medium. SD-4D: SD-Ade-His-Leu-Trp/X-α-Gal. The yeast culture was gradient diluted five times with sterilized water before being spread on the medium, and the gradient is represented as triangles.
